# Controllable Design of Polyamide Composite Membrane Separation Layer Structures via Metal–Organic Frameworks: A Review

**DOI:** 10.3390/membranes14090201

**Published:** 2024-09-21

**Authors:** Yanjun Jia, Xiaowen Huo, Lu Gao, Wei Shao, Na Chang

**Affiliations:** 1 School of Environmental Science and Engineering, Tiangong University, Tianjin 300387, China; 2 School of Textile Science and Engineering, Tiangong University, Tianjin 300387, China; 3 School of Chemical Engineering and Technology, Tiangong University, Tianjin 300387, China; 4 State Key Laboratory of Separation Membranes and Membrane Processes, Tianjin 300387, China

**Keywords:** metal–organic framework, thin-film nanocomposite, membrane, reverse osmosis, nanofiltration

## Abstract

Optimizing the structure of the polyamide (PA) layer to improve the separation performance of PA thin-film composite (TFC) membranes has always been a hot topic in the field of membrane preparation. As novel crystalline materials with high porosity, multi-functional groups, and good compatibility with membrane substrate, metal–organic frameworks (MOFs) have been introduced in the past decade for the modification of the PA structure in order to break through the separation trade-off between permeability and selectivity. This review begins by summarizing the recent progress in the control of MOF-based thin-film nanocomposite (TFN) membrane structures. The review also covers different strategies used for preparing TFN membranes. Additionally, it discusses the mechanisms behind how these strategies regulate the structure and properties of PA. Finally, the design of a competent MOF material that is suitable to reach the requirements for the fabrication of TFN membranes is also discussed. The aim of this paper is to provide key insights into the precise control of TFN-PA structures based on MOFs.

## 1. Introduction

Membrane separation technology has attracted attention as an energy-efficient separation technology, especially for nanofiltration (NF) and reverse osmosis (RO) membranes, which can be separated by molecular sieving or surface charge effects with selective interactions with target molecules [1,2]. The TFC membrane currently stands as the predominant membrane architecture within the realms of the nanofiltration (NF) and reverse osmosis (RO) separation processes. This sophisticated membrane structure comprises three integral components [3]: (i) A robust nonwoven substrate that serves as the backing layer, conferring mechanical integrity to the composite film. (ii) An intermediate layer formed by ultrafiltration (UF) membranes, which are produced via phase inversion on the nonwoven backing. This intermediate layer acts as a reactive scaffold for the subsequent deposition of an ultra-thin, dense layer. (iii) A top layer consisting of a polyamide (PA) layer with a thickness ranging from tens to hundreds of nanometers, fabricated through the interfacial polymerization of biphasic monomers on the intermediate layer. It is the PA layer, functioning as the topmost stratum, that plays a pivotal role in the selective separation process by acting as a sieve during mass transfer. Consequently, the strategic manipulation of the PA layer’s structure is of paramount importance in optimizing membrane performance.

Conventional RO membranes are typically prepared from m-phenylenediamine (MPD)/TMC (1,3,5-benzotricarbonyl chloride) monomers through an IP reaction, while nanofiltration membranes are typically prepared from piperazine (PIP)/TMC systems. The main reason for the difference in separation accuracy is that the PA layer formed by PIP has a larger free volume than the PA layer prepared by MPD. The semi-aliphatic molecular chains of PIP/TMC-based polymers are softer, resulting in a looser PA network, while pure aromatic MPD/TMC-based PA chains are arranged more tightly [4]. From this, it can be seen that the RO and NF membranes based on TFC membranes do not have the pores typically expected of ultrafiltration membranes. The definition of their pore size is more due to the “gaps” formed by the irregular stacking of polyamide chains caused by the uncontrollability of interfacial polymerization [5,6]. The pore size of RO membranes is usually sub-nanometer in size, and these membranes are effective in removing not only particles, but also molecules such as bacteria, organic contaminants, dyes, and univalent and multivalent ions [7,8]. NF membranes are a new type of membrane material with separation accuracy between RO and UF; their effective pore size is about 0.5–2 nm, and thus they can reject small molecules of 200–2000 Da. [9,10]. Moreover, the charging effect of NF membranes can be used to retain inorganic salt ions. Among these, the rejection rate of monovalent ions is low, while the rejection rate of divalent and multivalent ions is much higher [11].

Over the past few decades, NF and RO processes have played a dominant role in the field of wastewater treatment and desalination. Nevertheless, in addition to the mutual constraints of solute rejection and water flux (trade-off), PA-based membrane materials are prone to contamination. Furthermore, there are still issues with low selectivity in the separation of multivalent salts, the precise separation of small molecules with similar molecular weights, and the separation of specific ions. TFN membranes are novel membrane materials that combine the advantages of nanomaterials and TFC membranes. Numerous studies have shown that introducing porous nanomaterials such as titanium dioxide [12,13], silicon dioxide [14], graphene oxide [15] (GO), and carbon nanotubes [16] into membrane structures to prepare TFN membranes can effectively improve membrane performance. Wei et al. [13] successfully prepared composite nanofiltration membranes with a high flux and high rejection rate (Na_2_SO_4_: 99.7%) by introducing aminated TiO_2_ during the interfacial polymerization process between PIP and TMC. Kang et al. [17] prepared defect-free ultra-thin PA layers by interfacial polymerization using GO as the substrate modification layer. The prepared membrane had high selectivity for divalent and monovalent ions and good solvent resistance. However, the aggregation of rigid nanomaterials and the compatibility between organic polymers and inorganic particles are the main challenges in preparing these membranes. Poor compatibility often leads to poor stability and defects in the membranes, thereby affecting their separation performance.

In comparison to other inorganic nanofilm-modified materials, MOF materials are relatively novel. Not only do they possess the common characteristics of traditional porous materials—including high porosity, a large specific surface area, and low density—but they also exhibit advantages such as chemical, pore size, particle size, and charge adjustability. They are used as excellent TFC membrane-modified materials. Compared with polymer microporous materials, such as microporous polymers (PIMs) and covalent organic frameworks (COFs), although they have good compatibility and processability, they suffer from aging and accelerated porosity loss due to their polymer properties. An MOF is a novel crystalline material constructed by metal ions and organic bridging ligands [18]. Within this framework, organic connections are considered as the “pillars” of organic SBUs (secondary building units), and metal centers are considered as inorganic SBUs, serving as “joints” in MOF structures. The utilization of varying metal centers and ligands facilitates the production of MOFs as molecular building blocks, allowing these materials to retain the inherent physical and chemical properties of inorganic nanomaterials while exhibiting flexibility comparable to that of organic microporous materials [19].

MOFs have received increasing attention due to their tunable composition, controllable structure, and pore size equivalent to the kinetic diameter of ions on the angstrom scale. Compared to conventional polymers, there are issues with aging and an accelerated loss of porosity. MOFs possess stable mass transfer channels, and even minimal doping of MOFs is sufficient to alter the separation performance of TFC membranes. Compared with other rigid porous materials, MOFs have both rigid crystal structures and polymer properties such as abundant chemical functional groups. Pore channels can not only be modified by ligand functional groups in terms of size, hydrophilicity/lipophilicity, and charge properties, but they can also be altered in terms of dimensions (1D, 2D, 3D) to satisfy different conditions for the modulation of the PA layer structure. However, the advancement of MOF-based membranes for liquid separation is still in its early stages and lacks proper control over the development of preparation strategies and modeling applications for liquid separation. As a result, MOF-based membranes are not yet capable of providing comprehensive solutions to specific liquid-separation problems. In order to accurately construct MOF-based TFN membranes for the effective removal/separation of target components from liquid mixtures, researchers are trying to formulate rational MOF-based membrane design strategies to further expand the application areas of MOF-based membranes in liquid separation.

**Figure 1:**
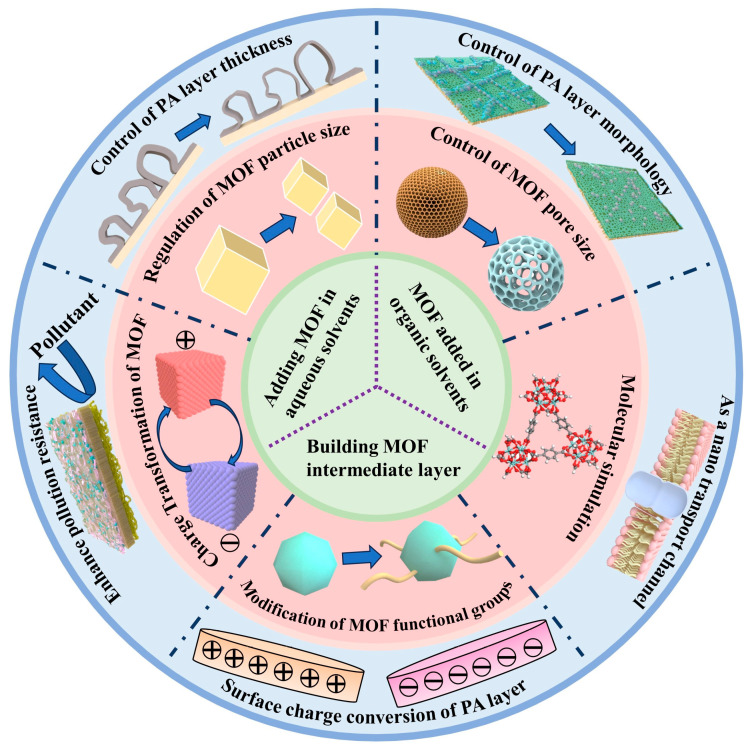
Schematic representation of the structural regulation of MOF and MOF-TFN membranes.

The recent literature indicates that the new dimension of “MOF-based TFN membranes” has now reached a stage where a comprehensive overview is necessary to summarize the latest advances, trends, and emerging opportunities in this area. Therefore, this review aims to provide an overview of the current developments in the field of MOF-based liquid TFN membranes. Different applications of TFC membranes entail varying requirements for membrane performance, particularly in terms of ion and salt permeability, as well as selectivity. As a result, customized designs of high-performance nanofiltration membranes are essential. However, this customization relies heavily on a thorough understanding of the effects of membrane fabrication methods and conditions on membrane performance, as well as the relationship between the physicochemical properties of the membrane structure and its performance. As shown in Figure 1, this paper firstly reviews the progress made in recent years in the controllable design of TFC membrane performance through the optimization of interfacial polymerization technology and the design of polyamide layer structures. Following this, the relationship between membrane structure and polymerization reaction is discussed based on the laws and mathematical model of the influence of modulated interfacial polymerization on polyamide structure. A specific scheme to adjust the membrane structure and physicochemical parameters by introducing MOFs to improve the membrane separation performance is also summarized. Then, the current and potential applications of these MOF-based TFN membranes in water treatment processes are systematically discussed by selecting appropriate additional strategies based on different separation requirements. Finally, this review emphasizes the prospects and challenges associated with customizing MOF-based TFN membranes for water treatment applications. This review establishes how MOFs are used to regulate the thickness, morphology, and nanochannels of PA layers, which is informative for designing polyamide layer structures using MOFs as a starting point.

## 2. Regulation of the Structure of the PA Layer by MOFs

To achieve a customized TFC membrane design with improved permeability and selectivity, two strategies can be implemented. The first approach is to modulate the IP process to achieve a controllable design of functional layer properties [20,21,22]. The other is to modulate the IP process by introducing other novel materials (such as MOFs) that are easily controllable in the reaction process to better tune the performance of TFC membranes [23,24,25]. This section follows these two strategies in writing. Firstly, it introduces the progress of researchers in recent years in regulating the thickness, morphology, nano-transport channels (pores or free volume), anti-pollution performance, and surface charge properties of the PA layer by influencing the IP process. Subsequently, the governing principles are distilled, allowing for the precise control of the IP process utilizing MOF materials, thereby facilitating the predictive regulation of the PA layer. Furthermore, our findings indicate that the application of MOFs for single-factor regulation of the PA layer structure frequently yields alterations in other properties of the PA layer.

### 2.1. Modulation of the Thickness of PA Layers

**Figure 2:**
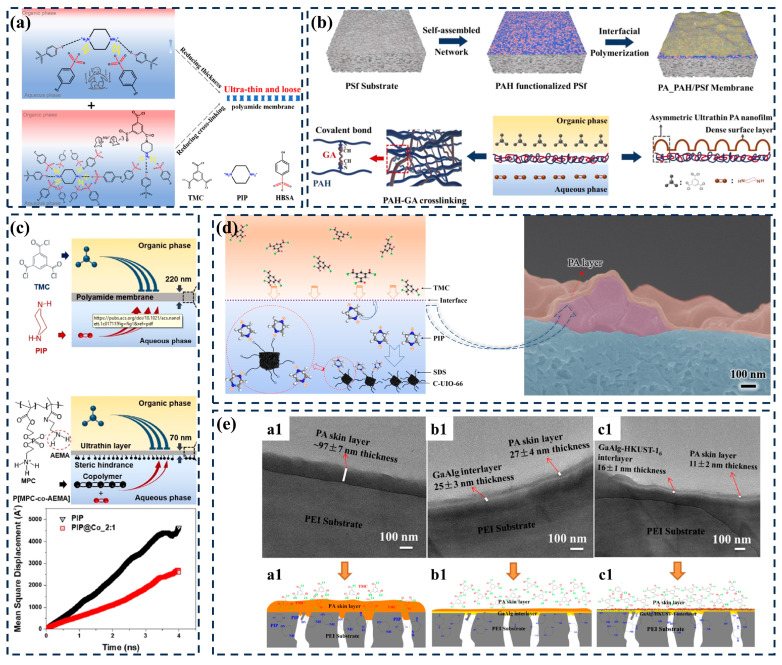
(**a**) Schematic of amine-decorated interlayer-mediated IP reaction and the mechanism of PAH-based interlayer for the formation of asymmetric PA nanofilm [29]. (**b**) Schematic diagram of the HBSA-regulated IP process and membrane structure [30]. (**c**) Schematic of the conventional IP and zwitterionic copolymer assembly-regulated IP, wherein the PIP molecules in the aqueous phase diffuse across the water/hexane interface to react with TMC in the hexane phase, with/without the hindrance of P[MPC-co-AEMA] [31]. (**d**) Explanation of the mechanism of C-UIO-66/SDS-coordinated regulation of interfacial polymerization [32]. (**e**) TEM cross-sectional images of PA/PEI (**a1**), PA/CaAlg/PEI (**b1**), and PA/CaAlg-HKUST-16/PEI (**c1**) [33].

Reducing the thickness of the separation layer and shortening the transport path of water molecules is undoubtedly an effective way to improve membrane permeability. According to numerous previous research reports, the thickness of TFC membranes is closely related to the diffusion rate of aqueous monomers [26,27,28]. As shown in Figure 2a, Zhang et al. [29] selected sodium 4-hydroxybenzenesulfonate (HBSA) as a modulator. Through the electrostatic interaction between HBSA and amine monomers, the diffusion of amine reaction monomers can be effectively slowed down, forming a super-thin-film structure. In addition, the uniform hydrogen bonding network around the amine monomer inhibits the IP reaction, resulting in a loose PA structure and a porosity nearly twice that of the original PA. The above two effects increase the permeability of water to 34.4 L·m^−2^·h^−1^ and the retention rate of Na_2_SO_4_ to 99.1%. Song et al. [30] used self-crosslinked polyaniline salt (PAH) as an intermediate layer for interfacial polymerization (IP). The self-assembled strong-charge and high-hydrophilicity ionomer network has a strong attraction to water amine monomers, increasing the heterogeneous energy barrier for diffusion to the organic phase and the polymerization of acyl chloride monomers, thus designing an asymmetric ultra-thin (8 nm) PA nanofilm (Figure 2b). The permeability of asymmetric PA membranes can reach 12.5 L·m^−2^·h^−1^·bar^−1^, which is three times higher than traditional PA membranes (4.4 L·m^−2^·h^−1^·bar^−1^). The Hideto team conducted a quantitative analysis for the first time on the diffusion rate of monomers and the thickness of the PA layer, and found that the thickness of the PA layer is directly proportional to one-third of the diffusion rate of amino PIP monomers into the organic phase (Figure 2c) [31]. In this regard, if a TFN membrane with an ultra-thin PA layer is prepared based on MOF materials, only the added MOF needs to be used as an aqueous additive to generate attractive forces with amine monomers to limit monomer diffusion. According to this pattern, our research group pre-deposited strongly negatively charged carbonized UiO-66 (C-UiO-66) nanoparticles modified with sodium dodecyl sulfate (SDS) onto the surface of the support layer. The diffusion rate of PIP to the organic phase was slowed down with the electrostatic attraction force, and a thinner PA layer (<33 nm, Figure 2d) than the original TFC membrane (144.29 ± 17.62 nm) was prepared [32]. Following the same regulatory approach, Li et al. [33] prepared a calcium alginate–HKUST-1 intermediate layer on a PEI support layer using an in situ growth method, and limited the diffusion rate of PIP through hydrogen bonding and covalent bonding (Figure 2e). The prepared TFN membrane had the characteristics of ultra-thin PA layer thickness, reduced from 97 nm to 11 nm, a large surface area (surface roughness increased from 75.6 nm to 113 nm), and high hydrophilicity (water contact angle reduced from 60.1° to 54.45°). Compared to other additives, MOFs, as diffusion inhibitors of aqueous monomers, not only reduce the thickness of the PA layer but also change the properties of other PA layers. This would be detrimental to the MOF’s single-factor regulation of the PA layer. For example, the appropriate pore size and larger specific surface area of MOF materials will provide low resistance channels for the transport of water molecules. A regular and uniform outer surface morphology will increase the roughness of the PA layer and improve the effective penetration area. Notably, water stability emerges as a critical factor that warrants attention during the selection of MOF materials. Moreover, MOF materials used for interfacial polymerization also need to have a certain degree of alkali resistance stability to facilitate stable dispersion in alkaline diamine aqueous solvents.

### 2.2. Regulation of the Morphology of PA Layers

**Figure 3:**
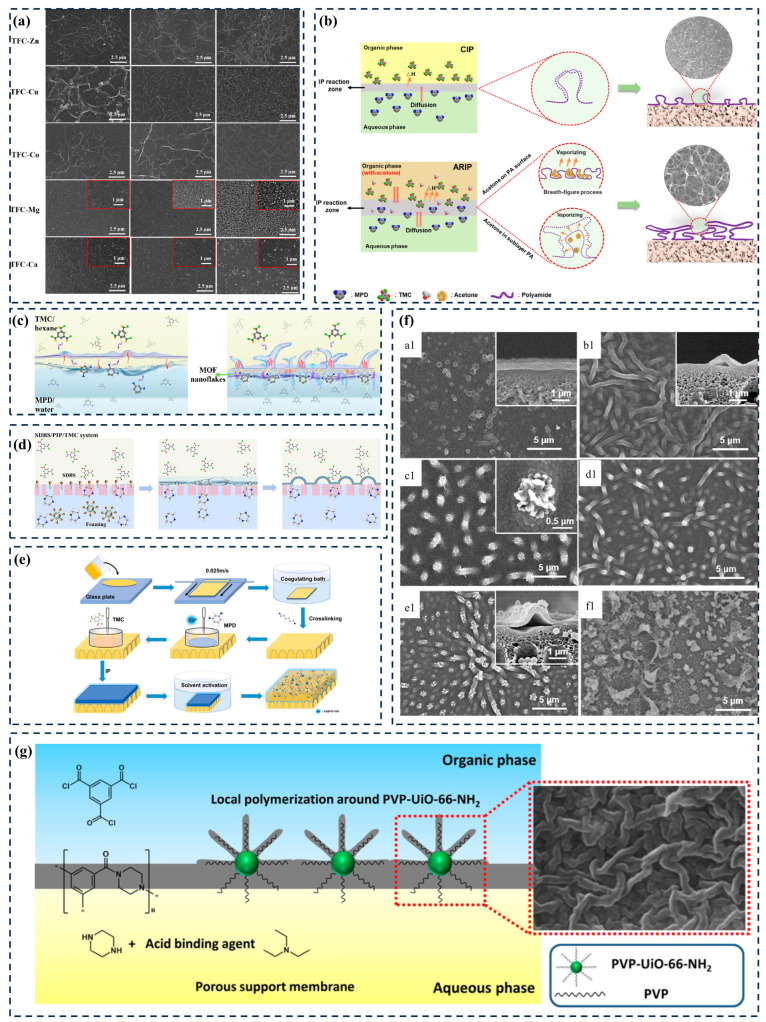
(**a**) Surface morphologies of TFC-M membrane modulated by preloaded metal nitrate solutions at varied concentrations: 40 mM, 60 mM, and 80 mM [35]. (**b**) Schematic pictures of polyamide layer formation in CIP and ARIP [36]. (**c**) Graphical illustration of the IP process at a free interface [free-interface polymerization (FIP)] [38]. (**d**) SDBS/PIP/TMC system for the formation of the crumpled TFC PA NF membrane [39]. (**e**) Schematic illustration for TFC-Cu membranes [42]. (**f**) Surface and cross-sectional SEM images of PA-pristine (**a1**), PA-PVA (**b1**), PA-flower1 (**c1**), PA-flower2 (**d1**), PA-flower3 (**e1**), and PA-flower4 (**f1**) membranes [43]. (**g**) Schematic diagram of synthesizing PVP-UiO-66-NH_2_ and preparing PVP-UiO-66-NH_2_@polyamide membrane [44].

The regulation of the morphology of the PA layer on the surface of TFC membranes is considered as a way to increase the effective permeation area and thus improve membrane flux. At present, the preparation of PA layers with “wrinkled” morphology can be roughly divided into two paths. Firstly, by affecting the diffusion of two-phase monomers, the phenomenon of “local activation and lateral inhibition” is generated, thereby driving diffusion instability to form a “Turing” structure as a representative case [34]. For example, Zhang et al. [35] regulated the interfacial polymerization between PIP and TMC by preloading divalent metal nitrates onto a polysulfone (PSF) carrier. Research has found that when the main group cations are Mg (II) and Ca (II), the water flux of the nanofiltration membrane can be increased by increasing its surface nanoscale nodular array without changing the pore size and adsorption properties of the membrane. When the transition metal cations were Zn (II), Cu (II), and Co (II), not only could a nanoscale longitudinal network with high surface area be formed to increase the water flux of the nanofiltration membranes (Figure 3a), but also the pore sizes of the prepared nanofiltration membranes were adjustable within the range of 0.54~0.90 Å, showing different selectivity and rejection capacities for inorganic salts and organic dyes. As shown in Figure 3b, Fu et al. [36] prepared high-performance TFC-PA OSN membranes on porous polyketone (PK) carriers by modulating IP with acetone as an organic cosolvent. The experimental results and molecular dynamics (MD) simulation analyses confirmed that the addition of acetone increased the width of the IP reaction zone, promoted a more intense exothermic reaction, and intensified the vaporization of acetone, resulting in the formation of a three-dimensional honeycomb structure with an ultra-high specific surface area. Compared with the TFC-PA membranes prepared by the conventional IP method, the methanol solvent permeability (7.0 LMH/bar) of the TFC-PA membranes prepared by the acetone-conditioned IP method was increased by a factor of 2.6, and the retention rate of methyl orange remained unchanged at 94.6%.

Secondly, rigid nanomaterials [37,38], surfactants [39], and salts [40,41] can be used as polymerization templates to introduce interfacial polymerization reactions to prepare PA layer structures with nano-bumps. For example, Wen et al. [38] arranged MOF nanosheets horizontally at the water/hexane interface and prepared an ultra-thin PA layer with a wrinkled surface morphology by accelerating the transfer of diamine monomers at the interface and retaining bubbles and reaction heat in the reaction zone. The inherent thickness of the layer was ~5 nm, and the crosslinking degree was as high as ~98% (Figure 3c). Qiu et al. [39] used sodium dodecylbenzene sulfonate (SDBS) to make an interface foam so that additional nano-bubbles could be introduced into the PIP/TMC system to shape the PA structure (Figure 3d). He et al. [42] used IP technology to prepare a TFC membrane with ridge valley morphology by doping copper (II) ions into the aqueous phase. The complexation of copper (II) ions with N atoms on meta phenylenediamine (MPD) affects the diffusion of MPD from the aqueous phase to the oil phase, leading to instability in the IP process and the formation of ridge-like morphology on the membrane surface (Figure 3e). Finally, by simply dissolving the crystals in deionized water, BFS can be removed from the TFC-PA membrane, resulting in a much higher water flux (three to four times) without affecting retention performance.

From the above literature, it can be seen that both reducing the thickness of the PA layer and obtaining a wrinkled PA layer structure can be achieved by regulating the IP reaction process. Regarding the surface morphology of the PA layer regulated by MOFs, the inherent rigid structure of MOFs will provide a rougher reaction template for the formation of the PA layer. The diversity and chemical modifiability of organic ligands undoubtedly increase the possibility of affecting monomer diffusion. Song et al. [43] prepared a nanofiltration membrane with a PA layer morphology resembling a “three-dimensional flower” by filling MIL-101(Cr) nanoparticles in a polyamide layer (Figure 3f). The experiment showed that under the test conditions of 1000 ppm Na_2_SO_4_ solution and 1 MPa pressure, the water flux of the PA flower membrane was 63.36 L/m^2^·h, and the retention rate of Na_2_SO_4_ was 89.86%. Xiao et al. [44] loaded an aqueous dispersion containing PVP-UiO-66-NH_2_ onto the carrier through vacuum filtration and fused PVP-UiO-66-NH_2_ into a polyamide layer using traditional IP technology. The presence of PVP-UiO-66-NH_2_ induces “local activation and lateral inhibition” of the IP system, leading to the appearance of nano-Turing structures on the membrane surface. PVP-UiO-66-NH_2_ with uniform particle size was successfully prepared and used for PVP-UiO-66-NH_2_@PA Composite membrane (Figure 3g).

However, to date, there have been no reports on the quantitative relationship between the polymerization process and the morphology of the PA layer, which is unfavorable for theoretically predicting and controlling the morphology of the PA layer. For example, reducing the diffusion rate of aqueous monomers is beneficial for both the thickness of the PA layer and the formation of wrinkled morphology. However, it is uncertain how to maintain the original morphology while reducing the thickness of the PA layer, or how to maintain the stoichiometric balance without reducing the thickness of the PA layer while changing its morphology. In addition, in MOF-TFN membranes, ignoring the effect of the morphology of MOF particles themselves on the structure of the PA layer, whether the weak force between them and the two-phase monomers would play a role in the interfacial aggregation process and thus affect the morphology and structure of the PA layer is still unclear. Based on density functional theory (DFT), our research group constructed a Lewis acid–base interaction relationship between an MOF and two-phase monomers (PIP and TMC molecules), determined the weak interaction force between the two, and applied it to the surface morphology control of the PA layer [45]. In summary, in subsequent research, we should deepen the exploration of the weak interaction force between MOFs and monomers, which cannot be ignored in terms of the impact on crosslinking degree, surface morphology, and the thickness of the PA layer.

### 2.3. Construction of Nano-Transmission Channels

**Figure 4:**
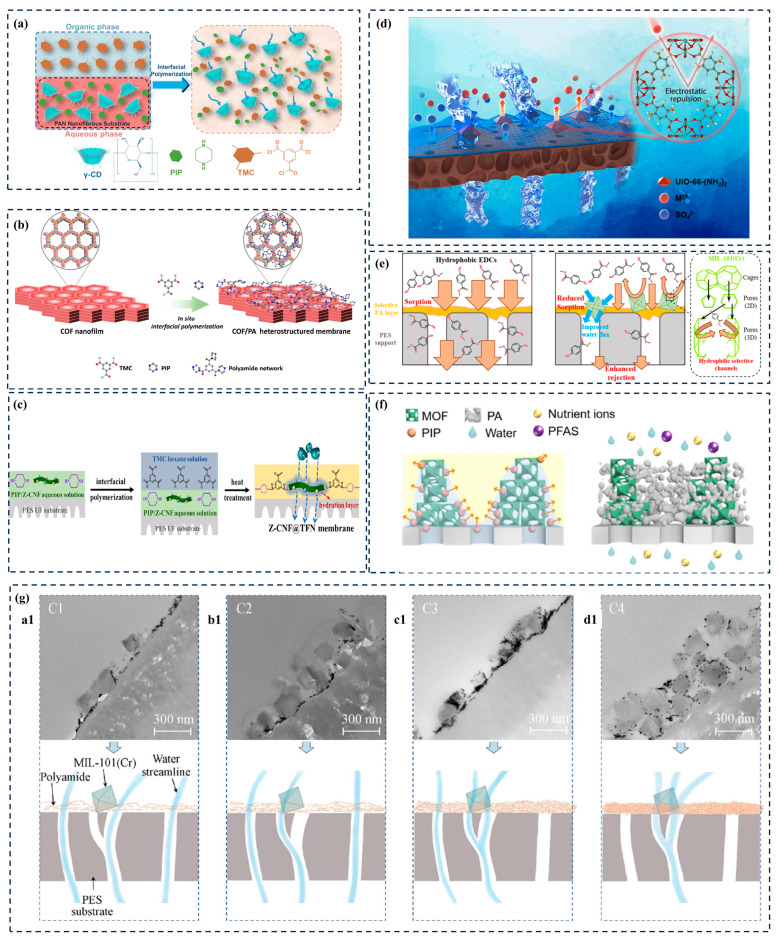
(**a**) A scheme illustrating the preparation of γ-CD-modulated TFNC NF membranes [47]. (**b**) Schematic illustration of the preparation of COF/PA hetero-structured membranes [48]. (**c**) Schematic illustration for the preparation process of Z-CNF@TFN membranes [49]. (**d**) Schematic diagram of ion rejection mechanism by TFN-Ds [50]. (**e**) Schematic diagram of the mechanism of enhanced rejection of EDCs by MIL-101(Cr) in the polyamide layer: EDC rejection by the control membrane; EDC rejection by the modified membrane [51]. (**f**) Schematics showing the transport (red arrows) of PIP molecules with gradient distribution across the water (blue)–organic (yellow) interface during CAIP (**left**) and the MOF-PA composite structure of the CAIP-MOF active layer with gradient-crosslinked PA (**right**) [52]. (**g**) GNPs-TEM characterization and corresponding schematics of primary water transport pathways of MOF-TFN membranes with different monomer concentrations: (**a1**) C1 of 0.5 wt.%, (**b1**) C2 of 1.0 wt.%, (**c1**) C3 of 1.5 wt.%, and (**d1**) C4 of 2.0 wt.% PIP in water. The GNP solution (1.0 × 10^12^ particles/mL) was dosed into a dead-end filtration cell for dynamic filtration under 5.0 bar pressure. The accelerating voltage of TEM characterization is 120 kV [53].

In a typical IP process, amine monomers in aqueous solution diffuse into the organic solvent phase and react violently with acyl chlorides at the water/organic interface through the Schotten–Baumann reaction. This uncontrolled diffusion and rapid aggregation form a PA layer with a multi-scale non-uniform pore size. Due to the dense amorphous structure of polyamide itself, there is a trade-off between the permeability and selectivity of traditional polyamide filter membranes, which restricts the further development of filter membranes. Liang et al. [46] formed polyamide membranes by surfactant-regulated interfacial polymerization (SARIP) to achieve precise ion separation. Zhang et al. [47] used γ-CD and PIP as aqueous monomers to undergo the polymerization reaction with TMC to prepare a PA separation layer. The three-dimensional hollow structure of γ-CD will provide additional direct nanochannels to facilitate water transport (Figure 4a). Due to the poor film-forming ability of COF membranes, the preparation of continuous, defect-free, and adjustable-pore-size COF membranes remains challenging. As shown in Figure 4b, Zhang et al. utilized a loose polyamide network to mask defects in COF nanofilms, thereby preparing composite films with heterogeneous structures for the precise screening of small molecules [48]. Similarly, adding nanomaterials during the interface polymerization process to construct low-resistance interface nanochannels is also a convenient and effective strategy for preparing high-performance membranes. Xia et al. [49] reported a new TFN membrane, to which zwitterionic nanocellulose was added in situ during interfacial polymerization (Figure 4c). Due to its increased surface hydrophilicity and high surface area, as well as additional hydrophilic interface nanochannels, the resulting membrane not only exhibited higher permeability (14.4 L·m^−2^·h^−1^·bar^−1^), but also enhanced the removal rate of divalent salts (R Na_2_SO_4_ = 98.3%).

Based on the above elaboration, there are only two ways to increase or improve the nano-transport channels of PA layers. One is to regulate the original interfacial polymerization process and thus the structure of the formed PA layer; the other is to introduce porous nanomaterials to reduce the mass transfer resistance and increase the transport channels. MOFs with well-defined window sizes and tunable chemical properties can fulfill all the conditions for providing suitable nanochannels for the mass transfer process. Moreover, their diverse and controllable structure, uniform pore size, and rich adsorption sites have opened up a new pathway for the targeted interception of specific ions/molecules. Xu et al. [50] constructed a novel dual-charge film by doping positively charged UiO-66-(NH_2_)_2_ into negatively charged polyamide layers. Due to the customizable nanochannels of MOFs, UiO-66-(NH_2_)_2_ nanoparticles containing amino-rich nanochannels were prepared through a ligand substitution strategy (Figure 4d). Calculations based on density functional theory further confirmed that UiO-66-(NH_2_)_2_ could effectively reject Ca^2+^, and the optimized TFN-D5 membrane achieved excellent removal of both CaCl_2_ (96.07%) and Na_2_SO_4_ (90.62%). Dai et al. [51] incorporated a hydrophilic MOF into the polyamide layer to create water/endocrine-disrupting compound (EDC)-selective nanochannels to enhance the inhibitory ability of EDCs (Figure 4e). Using an MIL-101(Cr) MOF as the nanofiller, the water flux of the MOF-0.20 TFN membrane (MOF 0.20 *w*/*v*.% in n-hexane) was 2.3 times higher than that of the control membrane. However, in common practice, uncontrolled interactions between the MOF and the PA matrix result in most of the nanomaterials being embedded or buried under the active layer of the TFN membrane, which greatly limits the use of water-permeable nanopores or nanochannels. Zhao et al. [52] proposed a method to prepare metal–organic framework (MOF) PA nanocomposite films by utilizing MOF pore capillary-action-assisted interface polymerization (CAIP). By adjusting the PA-MOF interaction through capillary force, MOF nanochannels are effectively exposed on the membrane surface, forming a PA matrix with a high crosslinking gradient in the vertical direction (Figure 4f). However, the separation mechanism of TFN membranes based on MOF materials is still unclear, and problems still exist, including the following: (1) Compared with polyamide pores, MOF pores have lower resistance. Will they become the only path for water transport? (2) If the pores in polyamide are “looser” than those in the MOF, will water preferentially penetrate through the TFN membrane as the main channel? (3) The contribution rate of nanochannels inside and around MOF-TFN membranes with different polyamide properties to solute transport. As shown in Figure 4g, Wang Zhiwei’s team proposed that water transport nanochannels in MOF-TFN membranes include (1) pores in the polyamide layer, (2) pores in the MOF, and (3) channels around the MOF (polyamide–MOF interface) [53]. However, specific methods for improving these three types of nanochannels based on MOFs have not yet been proposed.

## 3. Regulation of the Properties of the PA Layer by MOFs

### 3.1. Alterations in Hydrophilicity

Membrane fouling constitutes a critical factor that significantly restricts both the application and longevity of membrane technology. It is caused by particles, organics, and microorganisms in the intercepted materials adhering to or forming a gel layer. Membrane pollution will lead to an increase in transmission resistance and energy consumption [54,55]. Unlike regulating the structure of the PA layer, based on the analysis of membrane fouling mechanisms, the improvement of membrane fouling resistance can be achieved by changing the hydrophilicity of membrane surface [56,57]. Currently, enhancements in the hydrophilicity of membrane surfaces can be realized by increasing the density of the hydrophilic functional groups present on the surface, a modification technique that typically entails accompanying changes to the membrane’s surface charge properties. Guo et al. [58] prepared an anti-pollution amphoteric ionic nanofiltration membrane with tunable surface charge by introducing N^+^ into a polyamide matrix. The resulting amphiphilic ion membrane had positively charged N^+^ and negatively charged COO^−^. Meanwhile, the concentration of p-xylene dichloride (XDC) could precisely adjust the surface charge to realize the enhancement of the membrane’s anti-pollution performance. Tang et al. [59] designed a novel loose PA nanofiltration membrane using a traditional interfacial polymerization process with piperazine-2-carboxylic acid (PIP-COOH) and TMC as the aqueous-phase and organic-phase monomers, respectively. The presence of unique electron-withdrawing carboxyl groups on the PIP-COOH monomers not only provided abundant negative charges on the membrane surface, but also greatly reduced the aqueous monomer reactivity, diffusion rate, and solubility in n-hexane, resulting in a smooth PA layer with a membrane flux recovery of about 99% and excellent antifouling properties. Hu et al. [60] systematically investigated the effects of ionic group types (-COOH, -NH_2_, and amphoteric ions) on membrane antifouling and anti-scaling performance. Amphoteric ion-modified membranes with different anions (carboxylic acid, sulfonic acid, and phosphate groups) showed little difference in antifouling performance against organic pollutants, but had a significant effect on scale-inhibition performance. Amphoteric ion-modified membranes have good antifouling performance against charged and electrically neutral pollutants due to their good hydrophilicity and neutrality.

During the interface polymerization process, various metal-based fungicides, carbon-based nanomaterials, silica-based nanomaterials, and hydrophilic nanomaterials are used to improve the anti-fouling performance of TFC membranes. Correspondingly, more detailed requirements have been put forward for these nanomaterials, such as particle size, loading amount, density of hydrophilic functional groups, and compatibility between nanomaterials and polymers. However, the questions raised have not been well answered. It has been shown in numerous reports of TFN membranes that MOFs have relatively good modifiability. For example, Gao et al. [61] prepared a negatively charged loose NF membrane by incorporating hydrophilic TA@ZIF-8 nanoparticles into a PEI/TMA thermal crosslinking system. The membrane showed good antifouling properties for dyes (MB flux recovery of 98.5%). Cheng et al. [62] successfully prepared NF membranes by a constrained interfacial polymerization (CIP) method using MOF nanosheets as building blocks, achieving a gradual transition from two-dimensional (2D) MOF membranes to polyamide NF membranes. The enhanced antifouling performance of the prepared membranes was attributed to the increased surface hydrophilicity and negative charge of the polyamide after CIP in 2D MOF membranes, as well as the increased densification of the membranes, which reduced the adsorption of contaminants via inter-nanosheet defects. In fact, there are many variables that can affect the performance of the PA layer matrix, from nano-modified materials to the preparation of anti-fouling TFN membranes. For example, the pore size, porosity, and surface hydrophilicity of nanomaterials, as well as the roughness, thickness, and crosslinking degree of the PA layer have an effect. Analyzing the impact of changes in a single factor on the performance of the PA layer may lead to contradictory conclusions and inferences. Therefore, in the future, while ensuring high reproducibility of experiments, more systematic research will be conducted to explore the relationship between MOF modification and TFN membrane fouling prevention.

### 3.2. Alterations in Surface Charge Properties

**Figure 5:**
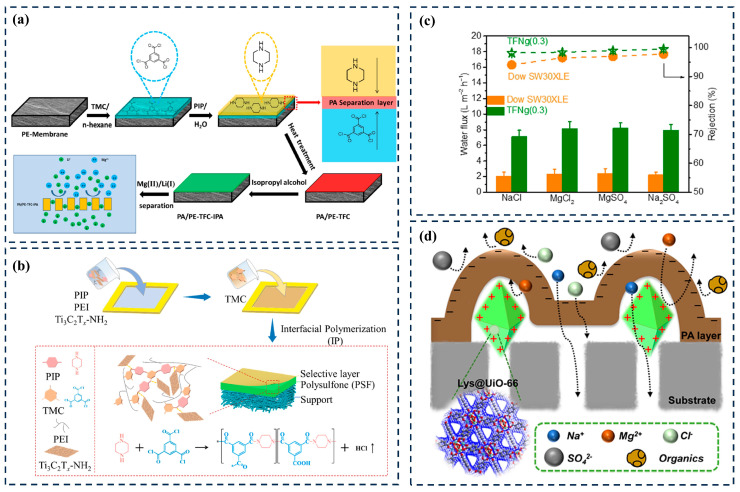
(**a**) Reverse-phase IP process for the preparation of PA/PE-TFC-IPA NF membranes [65]. (**b**) Schematic preparation process of NF membrane [66]. (**c**) Flux and retention performance of MIL-101 (Cr)-NH_2_-based TFN membrane [67]. (**d**) Proposed separation mechanism of TFN-LDU membranes [68].

The use of lithium batteries is rapidly increasing the consumption of lithium energy, and brine is one of the sources for extracting lithium. In China, saltwater is mainly composed of magnesium sulfide or chloride, which leads to the mixing of lithium (Li^+^) ions with other ions. Especially in high-magnesium–lithium-ratio (40–1837) saltwater, the separation of magnesium (Mg^2+^) and Li^+^ is challenging [63]. Li^+^ and divalent Mg^2+^ have similar chemical properties, with hydration radii of 0.382 nm (Li^+^) and 0.428 nm (Mg^2+^), respectively. On the other hand, Mg^2+^ carries twice as much charge as Li^+^, which is an entry point for designing NF membranes that effectively separate Li^+^ and Mg^2+^ [64]. According to Donnan’s exclusion theory, most positively or negatively charged NF membranes have a higher degree of repulsion towards divalent covalent ions, while their repulsion towards divalent counter ions is relatively low. In this case, NF membranes are undoubtedly a feasible Li^+^/Mg^2+^ separation method. Polyethylenimine is widely used as an aqueous-phase monomer for IP processes to prepare positively charged NF membranes. In contrast, the membrane surfaces in traditional PIP system NF show more negative charge. Then, how to make PIP-NF membrane surfaces realize positive charge transformation so as to accomplish the retention of divalent cations has become a difficult point in current research. Wang et al. [65] prepared polyamide/polyethylene composite thin-film (PA/PE-TFC) nanofiltration membranes by reversed-phase interfacial polymerization (RIP) to improve the separation performance of Mg (II)/Li (I) (Figure 5a). The PA/PE-TFC-IPA NF membranes formed by RIP with a high PIP concentration (0.3 wt.%) showed high MgCl_2_ retention (94.3 ± 0.7%) and a high Mg(II)/Li(I) separation coefficient when the TMC concentration was kept at 0.15 *w*/*v*.%. Li et al. [66] prepared a novel positively charged PA nanofiltration membrane using amino-functionalized Ti_3_C_2_T_x_ nanoparticles (Ti_3_C_2_T_x_-NH_2_, TN). The newly developed PA-TN membrane has a removal rate of over 96% for Ca^2+^ and Mg^2+^, and even in saline solutions, its flux is 2–3 times higher than many similar NF membranes reported in the literature (Figure 5b).

In summary, it can be seen that if the shift from negative to positive surface charge in TFC membranes is realized, more -NH_2_ functional groups should be exposed on the surface to mask the effect of COOH groups due to the hydrolysis of TMC molecules. Taking this as a starting point, Han et al. [67] successfully prepared highly selective TFN membranes by utilizing the post-synthesis modification of (3-glycerooxypropyl) triethoxysilane (GPTES) and trimethyl chloride (TMC) on MIL-101(Cr)-NH_2_ MOF-filled nanoparticles. In addition to this, the construction of positively charged nano-transport channels likewise provided an effective way to trap cations (Figure 5c). Gu et al. [68] used a pressure-assisted method to control the localization of lysine (Lys)@UiO-66 on a polyethersulfone (PES)-based membrane, and then prepared it through the interfacial polymerization of a Lys@UiO-66 thin-film nanocomposite membrane (TFN-LDU, Figure 5d). Uniformly distributed hydrophilic MOFs on the substrate induce the formation of regular tent-like and network topology polyamide structures. The load density is 17.13 μg cm^−2^. The permeability of the TFN-LDU8 membrane with g cm^−2^ increased by 55% (18.27 L·m^−2^·h^−1^·bar^−1^). Importantly, the TFN-LDU8 membranes exhibited a high rejection of divalent cation salts (97.81% for MgCl_2_ and 92.81% for CaCl_2_) and improved mono/divalent cation selectivity due to the pore resistance and electrostatic repulsion of Lys@UiO-66. Thus, for MOF-based TFN membranes, charge transformation modification seems to be solved by introducing positively charged nanochanneled MOF materials. The modification of MOF charge properties is discussed in detail in Section 4.3. As for the charge modulation of MOF-TFN membranes, the following points still need to be noted: (1) The introduction of MOF materials should not affect the original interfacial polymerization process, i.e., the addition of MOF will not lead to an increase in the number of COOH functional groups in the PA layer but rather an increase in the number of positively charged functional groups. (2) When preparing TFN membranes with positively charged surfaces, attention should be paid to the addition of MOF additives at the position of the PA layer. As is well known, when MOF is used as an aqueous phase additive, it will be located below the PA layer, while when MOF is used as an organic phase additive, nanoparticles will tend to exist on the surface of the PA layer. Under the premise of ensuring the structural integrity of the MOF, the MOF as an organic-phase additive is more conducive to the exposure of the prepared PA layer than MOFs containing positively charged functional groups to realize the charge conversion on the membrane surface.

Guided by the theory of regulating the interfacial polymerization process, this section discusses the basic principles of PA layer regulation, as well as the means of PA layer regulation using MOF materials to achieve the same effect. Through the analysis and discussion of existing studies, several points for future attention are drawn: First, simple and low-cost blending methods should be developed so that they can be adapted to the current TFN membrane fabrication process, and thus a controllable, scalable, and cost-effective MOF-TFN membrane fabrication process can be established. In addition, the economic benefits of using MOF-TFN membranes must outweigh the additional costs of development and production. Lastly, a long-term evaluation framework for assessing the separation performance of membranes should be instituted to quantify the degradation of membrane lifespan.

## 4. Strategy for the Preparation of MOF-TFN Membranes

Currently, the introduction of MOFs into the IP process is limited to aqueous-phase additives, organic-phase additives, and the construction of an intermediate layer between the support and PA layers. The rational design of the preparation strategy for MOF-based membranes is the key to achieving their precise and rapid liquid separation. Due to the sub-nanometer pore size and reticulated porous structure of MOFs, their role in the PA layer is similar to that of a water channel, i.e., promoting the permeability of water while blocking ions and small molecules of larger sizes. Thus, they can enhance permeability without affecting selectivity. Although the doping of MOFs is based on the blending process, the preparation process involves interfacial reactions between the aqueous and organic phases and cannot be considered a simple physical blending method. Consequently, the influence of MOFs on the diffusion and chemical interactions of monomers during interfacial polymerization will lead to changes in the membrane structure. It is not difficult to find that the PA layer structures prepared by dispersing MOFs in aqueous- and organic-phase solvents in previous studies are very different, including in terms of the position of the MOF relative to the PA layer, the crosslinking degree of the PA layer, and the thickness [69,70,71]. The proper selection of MOFs and the optimization of particle loading are equally important for designing TFN membranes with better performance and reducing the amount of nanofiller needed.

Reasonably designing the preparation strategy for MOF-based TFN membranes is the key to meeting their precise and rapid liquid separation requirements. The design principle of MOF-based high-efficiency liquid-separation membranes emphasizes “designing for the purpose”. For example, we must consider how to reasonably select MOFs based on the target components in liquid mixtures, what is the binding type between MOFs and membranes, Influence law of IP process on PA layer structure, and the advantages of MOFs in liquid separation. Compared to the trial-and-error method, the rational design process of MOF-based liquid-separation membranes (i.e., TFN membranes) aims to improve their separation ability through structural design, the reasonable classification of construction, and the optimization of performance characteristics based on selecting appropriate preparation methods and proposing reasonable design strategies. Currently, the strategies for incorporating MOFs into MOF-TFN membranes can be broadly categorized into three types: aqueous-phase additives, organic-phase additives, and the fabrication of MOF intermediate layers.

### 4.1. Regulating Monomer Polymerization Behavior—MOFs as Aqueous-Phase Additives

Since nanomaterials dispersed in aqueous-phase solvents are directly involved in the IP process and become embedded inside the PA matrix, they can significantly change the properties of the PA layer. Depending on the physicochemical properties and loading of the nanomaterials, the membrane properties can be positively or negatively changed in several ways. On one hand, nanomaterials within the PA layer can influence the crosslinking density of the active layer by interfering with the IP reaction of the monomers, leading to variations in membrane pore size and surface charge density. On the other hand, the introduction of nanomaterials generates additional water transport channels through the interfacial voids between the nanofillers and the PA matrix, and the intrinsic pores of some porous nanomaterials can also be used as fast channels to enhance water permeability. However, if the distribution of nanomaterials is non-uniform or if agglomeration occurs, the active layer of the membrane may produce non-selective defects, which inevitably affects solute selectivity. To improve the low compatibility between inorganic MOFs and flexible membrane matrixes, researchers have also attempted to enhance compatibility through MOF chemical modification. Zhang et al. [72] reported a TFN preparation strategy based on the synergistic interfacial reaction of Zr-MOF-UiO-66-NH_2_ with natural glucose to prepare biopolymer-based membranes. Due to the presence of glucose, the UiO-66-NH_2_ nanocrystals uniformly adhered to the 3D selective layer via an interfacial polymerization reaction, and the adhesion interaction of PDA provided good structural stability for the composite membrane. Although the IP reaction was able to anchor some nanofillers, their random distribution within the polyamide layer and limited loading made it difficult to maximize their benefits. Zhu et al. [73] attempted to achieve the controlled loading of hydrophilic MOF (UiO-66-NH_2_) by a simple vacuum filtration method. Specifically, an aqueous solution containing piperazine and homogeneously dispersed UiO-66-NH_2_ was transferred to the membrane surface by vacuum filtration, followed by IP with TMC. Covalent bonding between the terminal amine group of Zr-MOF and TMC allowed stable fixation of the MOF crystals in the formed PA layer. The localized MOF nano-aggregates acted as robust nodes connected by a continuous striped membrane formed after the IP reaction, forming a rough fishnet-like surface with a larger surface area than the nodular structure. This unique structure facilitates the creation of increased water transport channels without affecting salt rejection. Cui et al. [74] directly used Zr-MOF with amino groups as an aqueous-phase monomer to prepare flexible films by interfacial polymerization with TMC. The prepared composite films exhibited ultra-fast water transport performance (55.9 L·m^−2^·h^−1^·bar^−1^) with ultra-high MOF loading (55.8% wt.%). Although MOFs are attractive as aqueous-phase additives for PA layer performance enhancement, the alkalinity of aqueous MPD or PIP solutions limits the use of most high-performance MOFs. In recent years, some MOFs have been used as precursors to prepare size-controllable derivatized carbon materials that have been widely used in adsorption [75,76], catalysis [77], and electrochemistry [78], while maintaining the original morphology and structure. Cabello et al. [79] used a zirconium metal–organic skeleton (UiO-66) as a precursor to prepare porous carbon through a direct carbonization step, and the resulting porous carbon material was used to prepare poly(vinylidene fluoride) carbon composite membranes after etching treatment using hydrofluoric acid. Therefore, MOF-derived carbon materials are expected to solve the problems of MOF water stability and acid–base susceptibility to collapse.

### 4.2. Regulating the Surface Properties of the PA Layer—MOFs as Organic-Phase Additives

Different from aqueous-phase additives, TFN membranes prepared with MOFs as organic-phase additives tend to have a larger roughness but limited water flux enhancement, and the membrane surface morphology usually exhibits the granular structure of the added MOF so that the surface chemistry is mostly similar to that of the added MOF. In addition, due to the difference in the diffusion rate of the monomers in the two phases and the specificity of the PA layer formation location, the effect of MOFs on interfacial polymerization is relatively small; therefore, the TFN membranes prepared with MOFs as organic-phase additives usually do not modulate the interfacial polymerization process (and thus do not act on the PA layer structure), but rather construct specific solvent mass transfer channels from the point of view of the modulation of the properties of the MOF itself. For example, inspired by the Janus membrane concept, Dai et al. [80] grafted ethylenediamine (ED) onto the coordinating unsaturated metal sites inside MIL-101(Cr) to prepare MOFs with dual-charge properties (negatively charged surfaces and positively charged internal channels) to enhance the inhibition of positively and negatively charged PhACs^+^. In addition to the surface carboxyl groups repelling PhACs^+^, the positively charged internal water channels provide a strong barrier to PhACs^+^. On this basis, in order to reduce the non-selective defects of the MOF with the membrane matrix and increase its dispersion in organic solvents, Wang et al. [81] employed octadecylamine (ODA) to hydrophobically surface-modify the ZIF-67-derived nanocages to form ODA-h-NCs, which were applied to the PA layer of TFN membranes. Zhao et al. [82] found that, with the MOF-808 nanoparticle addition, the surface morphology of the resulting TFN membrane evolved from a nodular structure to a particle aggregation structure with increased roughness and enhanced hydrophilicity. It was also found by molecular dynamics simulation that the introduction of MOF-808 filler could significantly enhance the water transport in the TFN membrane. However, in the traditional interfacial polymerization process, in order to ensure that the acyl chloride group is not hydrolyzed, the selection of organic-phase solvents is basically limited to alkane solvents such as n-hexane and cyclohexane. Nanoscale MOFs cannot disperse well in such weakly polar and pure oil solvents, which is unfavorable for the preparation of well-dispersed MOFs and defect-free TFN membranes. Therefore, the preparation of MOF particles with good solvent dispersion in the organic phase is necessary if the limitation of MOF addition is to be broken through and thus the membrane flux is to be significantly enhanced. In addition, optimizing the interfacial polymerization step seems to be one of the effective ways to solve the agglomeration of MOFs in the organic phase. For example, the use of secondary polymerization as well as layer-by-layer self-assembly to preload the MOF particles onto the support layer can avoid the agglomeration of the MOF in the organic phase.

### 4.3. Avoiding Interface Defects—MOFs as Intermediate Layers

Due to the limitation of rigid nanoparticles, MOF nanoparticles inevitably agglomerate and cause interfacial defects. The introduction of an intermediate layer on the porous substrate prior to the IP process has recently been recognized as an effective way to reconcile the IP process and the PA layer structure, and is expected to break the traditional “trade-off” effect. Compared with the conventional IP process, the introduction of nanoparticles into the TFN membrane structure by preloading not only cleverly avoids the problem of particle agglomeration, but also increases amine storage and controls amine diffusion in the reactive interface by the presence of an intermediate layer, which facilitates the formation of an ultra-thin, defect-free, dense PA reactive layer, and results in a highly permeable membrane with a good solute rejection rate. This opens up a new dimension in the optimization of the structure and properties of TFN-PA membranes. In addition, the interlayer can be rationally designed with different types, structures, and chemical functions, thus making it possible to precisely control the IP reaction [83]. Wu et al. [84] used two different strategies to introduce ZIF-8 nanoparticles onto the surface of PSF substrates: one-step blending and two-step in situ growth. These strategies resulted in a certain number of ZIF-8 nanoparticles being embedded in the polyamide active layer or covered by the polyamide layer, respectively. The role of ZIF-8 nanoparticles exposed to the membrane surface under both preparation strategies was elucidated by characterizing the morphology, physicochemical properties, and interception properties (salt and dye) of the TFN membrane. However, the dispersion of MOF nanomaterials on the support layer remains a challenge. The electrophoretic deposition (EPD) mode immobilization MOF strategy was applied as a potentially viable option for the preparation of TFN membranes. Li et al. [85] achieved the transfer of ZIF-8 nuclei onto UF substrates by adjusting the aging and deposition time. Subsequently, a vacuum-assisted IP reaction was carried out to prepare nanofiltration membranes using piperazine (PIP) and trimethyl chloride (TMC) as monomers.

From the above literature, it is not difficult to see that there is a shortcoming in the use of MOFs as intermediate layers to regulate the structure of the PA layer: the MOF-to-MOF force is weak, and there is no bonding between the MOF and the substrate. Thus, it is prone to MOF leakage. Instead of expressing it as an intermediate layer, the description of preloading seems to be more accurate, which is due to the fact that there is no continuous MOF phase on the substrate of the support layer. In situ growth may be a solution to prepare “seamless” MOF layers; for example, Xu’s team has published successive papers on the preparation of ZIF-8 thin films with excellent divalent salt retention via in situ growth and an LBL strategy using a plant polyphenol tannic acid (TA) layer as an intermediate layer on top of a PES support layer [86,87]. However, unlike the preparation of gas separation membranes, the in-situ growth method for preparing TFN membranes requires particularly high requirements for MOFs, and the preparation of water stable MOFs and mild conditions for MOF synthesis (room temperature, room temperature) undoubtedly increase the development resistance of this method [88,89,90].

## 5. Optimization of the Properties of MOFs

The structure and performance of MOFs largely determine the separation performance of TFN membranes. From the perspective of rational design, it is proposed that an ideal MOF-based membrane should have efficient separation capability, long-term operation capability, and satisfactory component stability, while its preparation process should be as environmentally friendly as possible. According to this viewpoint, the basic principle of screening MOFs should be to utilize their structural advantages as criteria while meeting the requirements for separation membranes in different liquid-separation applications. For the rational selection of MOFs, it is imperative to consider their dispersibility and stability, as well as surface chemical and charge properties.

### 5.1. Modulation of MOF Particle Size to Address Dispersion Issues

Particle size is an important parameter that affects particle-selective layer interactions, membrane morphology, surface roughness, and separation properties. In a previous study on the effect of zeolite crystal size on zeolite–polyamide TFN membranes, it was shown that smaller zeolites have stronger permeability enhancement, but larger zeolites have more favorable surface properties [91]. Based on this theory, He et al. [92] synthesized three water-stable MOF UiO-66 nanoparticles with different diameters (30, 100, and 500 nm) and doped them into selective layers to form TFN membranes. Among the three TFN membranes, the TFN membrane consisting of 30 nm of UiO-66 showed the best performance. In addition, the TFN membranes containing 0.15 wt.% UiO-66 (30 nm) showed 96.5%, 97.4%, and 98.6% removal of SeO_3_^2−^, SeO_4_^2−^, and HAsO_4_^2−^, respectively, with the highest pure water flux of 11.5 LMH/bar. However, it is not the case that nanoparticles with smaller particle-size dimensions can produce a larger interfacial area with a continuous polymer matrix. Lee et al. found that the deposition of ZIF-8 nanoparticles on PSF carriers was influenced by particle size [93]. Based on classical deposition theory, the interfacial area of the ZIF-8/polyamide layer is influenced by the equilibrium relationship between the external surface area of individual particles and the surface coverage of the particle.

For MOF synthesis engineering, there are currently many methods for regulating MOF particle size. In addition to particle size control methods, such as adding initiator solvents [94], coordinating regulation [95,96], and changing precursor concentrations [97,98], Bunzen et al. [99] found that the temperature and heating rate during MOF synthesis can control the nucleation process and crystal growth, which contribute to the formation of nano-MOFs (NMOFs). However, previous studies have found that coordination regulation is the most common method. Tsuruoka et al. [100] found that controlling the interaction between metal ions and organic ligands, known as the “coordination equilibrium”, is crucial when changing the characteristics of MOF crystals. Therefore, the team regulates the coordination equilibrium by adding end-capping reagents (modulators) with the same chemical function as the ligands, preventing coordination interactions between metal ions and organic ligands, and thereby creating a competitive situation that regulates the rate of skeleton extension and crystal growth. Diring et al. used a combination of microwave-assisted solvothermal and coordination adjustment methods to control the size of porous coordination polymers (PCP, Cu_3_(btc)_2_). By adjusting the concentration of acid additives to precisely control the nucleation rate of the PCP framework and thus control the generated crystal size, homogeneous PCP nanocrystals with sizes ranging from tens of nanometers to several micrometers were successfully obtained [101]. Due to the almost identical composition and geometric shape of MOF-5 and IRMOF-3, Llabrés et al. explored the possibility of creating heterogeneous materials by using a mixture of the two ligands [102]. To illustrate the different growth modes of these two MOFs, a kinetic study was conducted in this experiment. Although the difference between the two dynamics did not seem statistically significant after the Kolmogorov–Smirnov experiment, it was predicted that the size of the MOF-5 crystal would be smaller than that of the IRMOF-3 crystal.

Based on the summary of previous works, several main mechanisms can be used to describe the control of MOF size [103]. The most effective ones are as follows: (1) the coordination modulation mechanism (also known as modulation formed by complexes); (2) the protonation/deprotonation mechanism (acid–base regulation); (3) the regulation of surfactants/end-capping agents. Usually, smaller nanomaterials are beneficial for fully utilizing the larger interface area between dispersed nanomaterials and continuous polymer matrices. However, due to Ostwald ripening, smaller nanoparticles are more prone to agglomeration, leading to non-selective defects in the membrane structure. Therefore, the influence of nanoparticle size should be appropriately considered together with the dispersion characteristics of nanoparticles.

### 5.2. Modulation of MOF Pore Size for Precise Separation of Small Molecules

The addition of MOF particles significantly increases the flux of TFN membranes, and once embedded in the polyamide matrix, these porous structures are considered to provide a preferred water transport pathway. Moreover, researchers believe that an appropriate window size helps to trap solutes, leading to higher membrane selectivity [104]. For example, Cseri et al. [105] used UiO-66-NH_2_, UiO-67-NH_2_, and UiO-68-NH_2_ with the same particle size and chemical morphology but different pore diameters as membrane-modified nanofillers. A nanofiltration membrane material utilizing MOF pore size for selective screening was successfully prepared by crosslinking amino groups in MOF ligands with PNIPAM chains to reduce the interfacial gap of MMM. Wang et al. [106] proposed a novel cobalt-based metal–organic framework (Co MOF) nanosheet film for efficient and selective dye recovery. Compared with non-porous nanosheet membranes, Co MOF membranes have superior permeability and selectivity, and their in-plane pore size can be precisely adjusted. By adjusting the length of the ligand, the in-plane pore size can be precisely adjusted from 1.01 × 0.63 to 1.43 × 0.64 nm^2^. Under diffusion- and pressure-driven filtration modes, Co MOF membranes exhibit high selectivity towards salt and dyes, as well as excellent tunable pure water permeability and high dye rejection.

An effective method for regulating the pore window of MOF materials is to change the ligand length. Yuan et al. [107] achieved the continuous control of the crystal cell size, surface area, and pore size of MOFs using different organic ligands and ligand ratios. The edge length of the crystal cell can undergo continuous and precise changes between 17.83 and 32.63 Å, with an adjustable BET specific surface area range of 585–3791 m^2^/g^−1^ and a maximum pore size of 15.9 Å. Another approach is to modify specific ligands with functional groups to change the pore size. Bonnett et al. [104] synthesized PCN-222 nanoparticles with myristic acid and modified them to adjust the MOF pore size for the preparation of TFN reverse osmosis membranes. Compared with the TFC membrane, a nearly 100% increase in flux was observed, while salt rejection remained above 95%. Therefore, MOFs with customizable pore sizes have great potential for constructing other ion channels and separation membranes.

### 5.3. Modulation of MOF Functional Groups to Address Matrix Compatibility Issues

The manufacturing of MOF-based TFNs can synergistically combine the advantages of polyamide and MOF components, providing additional degrees of freedom for customized membrane performance: nano-fillers can modify the free volume elements inside polyamide and may increase permeability and selectivity, while the wide range of polyamide maintains the basic mechanical stability of the membrane. When these particles are added to the polyamide layer, the most significant effect is an increase in permeability; however, this increase in permeability is accompanied by a more significant change in retention rate. Moreover, ideally, effective fillers should have high hydrophilicity to increase water absorption and reduce fouling. However, these hydrophilic nanoparticles typically need to be dispersed in hydrophobic organic solvents, such as hexane, to form a membrane through interfacial polymerization. Due to their high surface energy, nanoparticles tend to agglomerate in the organic phase, ultimately leading to low filler loading and the formation of non-selective voids between fillers in polyamide membranes. In addition, the poor compatibility and weak interaction between the filler and polyamide lead to interface defects and non-selective bypass phenomena during the separation process, which reduces the expected benefits of the filler particles. Han et al. [67] modified the surface of MIL-101(Cr)-NH_2_ particles with triethoxysilane, improved the dispersion of MIL-101(Cr)-NH_2_ particles in organic monomer solutions, prepared stable particle suspensions, significantly suppressed particle aggregation, increased particle loading in the polyamide layer, and did not form non-selective voids. The in situ chemical crosslinking method of MIL-101 (Cr)-NH_2_ with polyamide improved the compatibility between the polymer and filler, and eliminated interface defects. Similarly, when selecting MIL-101(Cr) as a water-stable MOF material, Han et al. [108] did not modify the MOF with functional groups to increase compatibility with the matrix, but instead grafted different ionic liquids (ILs) onto the coordination unsaturated sites (CUS) of MIL-101(Cr). The modified TFN membrane had good mechanical strength and flexibility, as well as improved screening efficiency and electrostatic repulsion against divalent ions. Compared with Mn, Co, and Ni, the selectivity of the modified TFN membrane for lithium increased by four times.

In order to obtain ideal MOF chemical structures and composite materials, various synthesis methods have been developed, including bottom–up synthesis, top–down methods, and post-synthesis modification. Among them, modification after synthesis has been widely studied, as the obtained MOF composite material will be endowed with new properties and functions while maintaining its topology and porosity. The modifier can be modified on the surface or inside the pores of MOFs through post-synthesis modification to obtain MOF composite materials. Usually, non-covalent interactions, coordination bonds, and covalent bonds are the main driving forces for connecting MOFs and MOF modifiers [109]. (1) Non-covalent modification: Non-covalent-bond-bound MOF-modified materials can maintain both the original structure of MOFs and the high activity of modifiers, with the most typical example being the preparation of MOF polymer composites. For example, Xue et al. [110] used a BUT-33 MOF material as a carrier and prepared an MOF polymer material with a high Au^3+^ adsorption rate via the self-polymerization of phenylenediamine in MOF pores. The removal rate of Au^3+^ in less than 45 s was over 99%. (2) Coordination bond modification: Zhang et al. [111] successfully prepared β-cyclodextrin using an environmentally friendly method, and then prepared the target product (β-CD-MOF) using solvent diffusion method (β-CD-MOF). (3) Covalent bond grafting: Cui et al. [112] constructed a high-loading MOF-based composite film: First, ultra-small and stable UiO-66-NH_2_ nanoparticles were connected to TMC, and a continuous UiO-66-NH_2_-TMC membrane was constructed through interfacial polymerization. The UiO-66-NH_2_-TMC membrane had a high MOF loading capacity (>57%) and abundant intergranular pores, which are conducive to Li^+^/Mg^2+^ separation. Functional groups play an important role in MOF chemistry, as their potential in interfacial polymerization processes largely depends on the possibility of integrating various chemical functional groups into MOFs. Functionalized MOFs can exhibit interesting physical and chemical properties. In addition, via post-synthesis modification, it is possible to insert a functional group into an MOF without significantly changing its lattice structure in order to improve its texture characteristics or enhance its application in organic matrices.

### 5.4. Design of MOFs with Different Charge Properties

The most widely accepted theory for the separation mechanism of nanofiltration membranes is the synergistic effect of the Donnan effect and pore sieving. Therefore, the charge properties of the membrane surface are closely related to its separation performance. Ji et al. [70] synthesized a series of compounds with different properties with ζ-UiO-66 potential, and these MOFs were added to the aqueous and organic phases for interfacial polymerization. With different ζ in the presence of MOFs with potential, TFN membranes with different PA layer morphologies were obtained. Of these, the more wrinkled PA layer structures provided a larger effective permeation area and enhanced water permeability. Our research group investigated the effect of MOF particle size and charge on the PA layer by regulating the particle size and charge of CAU-1. Then, we introduced it into a TMC/n-hexane solution to prepare TFN composite films based on MOF materials (CAU-1). The study found that MOF materials with strong positive charges can generate Turing structures in the PA layer, thereby increasing the flux [113]. It can be seen that when MOFs with different charge properties participate in interfacial polymerization, they usually have an impact on reducing the thickness and adjusting the morphology of the PA layer.

Here, we summarize two methods for regulating MOF charge: (i) preparing MOF materials with defects, including ligand deficiency and metal cluster deficiency [114,115]; (ii) the charge modification of MOFs [32,116] (functional group grafting, surfactant modification). Recently, Hong et al. [116] reported a strategy for preparing anionic pacs-type porous materials by combining pore space partitioning and charge redistribution. This method utilizes a negatively charged pore allocation ligand (2,5,8-tri-(4-pyridyl)-1,3,4,6,7,9-hexaazaene, H-tph) to achieve both pore allocation and charge redistribution.

## 6. MOF-TFN Membrane Design Guided by Molecular Simulation

Membrane separation technology, especially nanofiltration and reverse osmosis, has greatly developed in the past few decades. However, due to the uncontrollable nature of interface aggregation and rapid reactions, many scientific issues remain unresolved. Computer simulation is more suitable for the mechanism of interface polymerization at the molecular level, as it can provide microscopic or mesoscopic insights for understanding this dynamic process. In addition, in computer simulation, it is also possible to systematically and continuously adjust the control factors that greatly affect the interface aggregation results. Therefore, a detailed overview of the interface polymerization process can be clearly obtained through computer simulation strategies, and the underlying mechanisms of supramolecular polymer formation can be elucidated. For example, the pore size and free volume of the PA layer play a crucial role in water flux. Approximately 15–32% of the volume in the PA layer is occupied by nanopores, providing channels for water transport [117]. Zhang et al. constructed a PA layer through MD simulation, demonstrating the characteristics of real membranes and studying the composition structure permeability relationship of PA membranes at the molecular level [118]. Zhang et al. [119] used a non-equilibrium molecular dynamics (NEMD) method to simulate the water permeation and salt retention of PA membranes with different thicknesses and pore sizes. It was found that the small crosslinked pores (1.00–4.00 Å) of PA were mainly responsible for salt removal, while large stacked pores (4.00–6.00 Å) allowed for rapid water transport.

**Figure 6:**
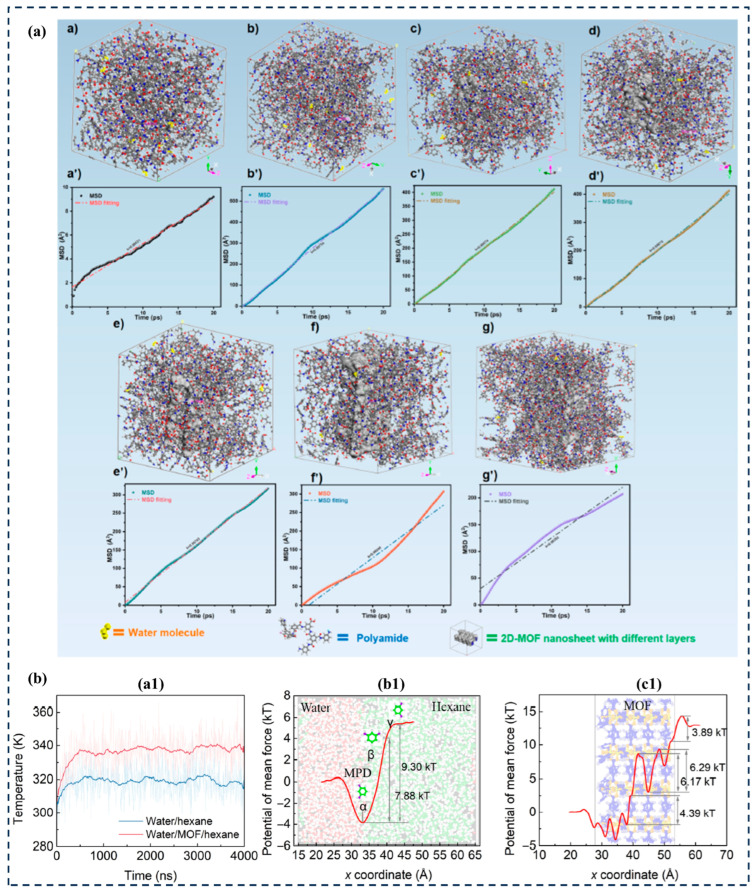
(**a**) Snapshot of water molecule diffusion inside various models. (**a**–**g**) The water molecule diffusion inside C-0, C-1, C-2, C-3, C-4, C-5, and C-6 models. (**a’**–**g’**) The MSD of water molecules inside C-0, C-1, C-2, C-3, C-4, C-5, and C-6 models. The numbers 0–6 represent the amount of 2D-MOF layer [120]. (**b**) (**a1**) Temperature evolution at the interface in the water/hexane system and water/MOF/hexane system. (**b1**) MD simulation of the potential of mean force with the MPD molecule at different locations along the x coordinate in the water/hexane system. (**c1**) MD simulation of the potential of mean force with the MPD molecule at different locations along the x coordinate for the water/MOF/hexane system [121].

However, molecular simulation of the formed PA layer alone is far from sufficient. For traditional interfacial polymerization reactions, it is necessary to form a film through the polymerization reaction at incompatible two-phase interfaces. The concentration of the two-phase monomers, solution interfacial tension, monomer diffusion rate, and zwitterionic additives all affect the polymer chain configuration (bulk density, free volume, etc.) and macroscopic structure (thickness, morphology, etc.) of the PA layer. Therefore, it is necessary to study the interface polymerization process through molecular dynamics simulation. Only a small portion of current research focuses on the interface polymerization process of MOFs to explore the formation mechanism of the PA layer. Liu et al. [120] used molecular dynamics simulations to investigate the additional pores and free water molecules of 2D MOF nanosheets added to the PA layer. The H bond between 2D MOF nanosheets coordinated with water can effectively promote the diffusion of water molecules (Figure 6a). Wen et al. [38] conducted MD simulations on the heat dissipation at the water/hexane interface, confirming that the presence of interfacial MOFs hinders the heat dissipation of polymerization reactions, reduces energy loss, and promotes the transfer of MPD through the interface (Figure 6b). However, the generation of the PA layer is a complex reaction diffusion process. Compared to pure TFC membranes, the molecular simulation of TFC membranes based on MOF materials is more difficult. Given that amine monomers in the aqueous phase must reach the water/hexane interface before reacting with TMC monomers in the organic phase, MOFs in the reaction system may affect the diffusion of aqueous monomers towards the interface. Moreover, because theoretically an infinite variety of MOFs can be designed using different combinations of metal and organic ligands, the exposed metal cation sites [121], ligand stretching [122], pore size, and different functional group modifications [123] will all affect the interfacial polymerization process. Further research on this aspect is needed. The performance of a membrane depends on its structure and physicochemical properties, including thickness, pore size, porosity, surface charge density, hydrophilicity, and roughness. Understanding the relationship between membrane performance and MOF structure, as well as the impact of MOF chemical properties on membrane performance, will help tailor composite membranes for different application fields.

## 7. Conclusions and Outlook

In terms of MOF structure design, to improve the dispersion and compatibility of MOFs in organic matrices, starting from material preparation, new MOF molecular designs with special functions, such as magnetic response, flexible MOFs, and “breathing” MOFs with adjustable pore size response, should be established. Among them, magnetic MOFs can be precisely positioned in the selection layer to design TFN membranes with ideal discrete MOF structures. The use of flexible MOFs in polymers undoubtedly adds to the compatibility of MOFs in organic matrices. The preparation of pH-responsive and pressure-responsive MOFs with adjustable pore channels provides a new approach for achieving functional filtration materials that meet different separation accuracies and requirements. Furthermore, there is a lack of research investigating whether the crystal plane properties of three-dimensional or two-dimensional MOFs significantly influence the interfacial polymerization process. There is still a lack of exploration on whether the regulation of crystal growth and the design of MOF dimensions will cause changes in membrane performance.

In terms of MOF-based PA layer regulation, although efforts have been made to develop design strategies for MOF-based membranes, some of the drawbacks of these methods may limit their potential applications. Therefore, in order to maximize the advantages of MOFs in membranes, it is highly desirable to develop a strategy that can seamlessly combine MOFs and polymers, thereby integrating the chemical and physical properties of MOFs with the mechanical and processing properties of polymers. In addition, the future application of MOF membranes in industrial production still faces challenges, and the long-term stability of MOF membranes in acidic/alkaline environments, complex organic solvent systems, and high temperatures needs further research.

Regarding molecular simulation, the synergistic combination of molecular simulation and advanced experiments may provide additional insights into the intrinsic relationship between the chemical properties of MOFs and the pore structure and separation performance of PA layers. At present, there are no relevant reports on the establishment of transport and separation mechanisms across MOF-TFN membranes, or the calculation of energy barriers for mass transfer processes through pores. In addition, due to the limitations of computational resources, the complete structural characteristics of MOFs cannot be reflected in simulation calculations due to their topological structure. Therefore, the calculation of the influence of MOFs on the diffusion rate of two-phase monomers still faces difficulties. In future research, efforts should be made to strengthen the development of new simulation calculations to adapt to molecular simulations in different scenarios.

## Data Availability

No new data were created or analyzed in this study.

## Abbreviations

**Abbreviation**	**Terminology**	**Abbreviation**	**Terminology**
PA	polyamide	TFC	thin-film composite
MOFs	metal–organic frameworks	TFN	thin-film nanocomposite
NF	nanofiltration	RO	reverse osmosis
UF	ultrafiltration	MPD	m-phenylenediamine
TMC	1,3,5-benzotricarbonyl chloride	PIP	piperazine
GO	graphene oxide	COFs	covalent organic frameworks
PIMs	inherently microporous polymers	SBUs	secondary building units
IP	interfacial polymerization	HBSA	sodium 4-hydroxybenzenesulfonate
PAH	polyaniline salt	SDS	sodium dodecyl sulfate
PEI	polyetherimide	PSF	polysulfone
PK	polyketone	MD	molecular dynamics
SDBS	sodium dodecylbenzene sulfonate	BFS	branching fractal structures
PVP	polyvinyl pyrrolidone	DFT	density functional theory
CIP	conventional interfacial polymerization	ARIP	acetone-regulated interfacial polymerization
SARIP	surfactant-regulated interfacial polymerization	γ-CD	γ-Cyclodextrin
EDC	Endocrine-disrupting compound	CAIP	capillary-action-assisted interface polymerization
XCD	p-xylene dichloride	piperazine-2-carboxylic acid	PIP-COOH
TA	tannic acid	PE	polyethylene
RIP	reversed-phase interfacial polymerization	GPTES	3-glycerooxypropyl triethoxysilane
PES	polyethersulfone	Lys	localization of lysine
PDA	polydopamine	ED	ethylenediamine
PhAC	pharmacological active compounds	ODA	octadecylamine
EPD	electrophoretic deposition	ILs	ionic liquids
CUS	coordination unsaturated sites	NEMD	non-equilibrium molecular dynamics
